# Recidivism Rates of Treated, Non-Treated and Dropout Adolescent Who Have Sexually Offended: a Non-Randomized Study

**DOI:** 10.3389/fpsyg.2021.757242

**Published:** 2021-10-13

**Authors:** Julie Carpentier, Jean Proulx

**Affiliations:** ^1^Department of Psychoeducation, UQTR, Université du Québec à Trois-Rivières, Trois-Rivières, QC, Canada; ^2^Department of Criminology, UdeM, Institut National de Psychiatrie Légale Philippe-Pinel, Montreal, QC, Canada; ^3^Université de Montréal, Montreal, QC, Canada

**Keywords:** adolescent, sex offender, treatment effect, recidivism, typology

## Abstract

The primary objective of this study was to evaluate the effectiveness of a cognitive-behavioral treatment in reducing recidivism by adolescents who have sexually offended (ASO). A secondary objective was to determine whether typologies based on victim age (child, adult/peer, mixed) and relationship (intrafamilial, extra familial, intra/extra familial) discriminate ASO in terms of response to treatment and recidivism. The sample comprised 327 adolescents 12–18 years old (*M* = 15.8 years, *SD* = 1.9) who were evaluated in an outpatient clinic after committing a contact sexual assault. Official data on recidivism (criminal charges) was collected after a follow-up period of 21–162 months (*M* = 7.8 years, *SD* = 32.2). Survival analysis indicated that adolescents who completed treatment (*n* = 62) had a recidivism rate for violence (including sexual violence) almost half that of adolescents who had either not completed the treatment or not received treatment (*n* = 261), (16.1 vs. 30.7%). Neither of the two typologies studied had any effect on the completion of treatment. However, sexual aggression against adults/peers was associated with an increased probability of violent re-offending. These results confirm the effectiveness of this cognitive-behavioral treatment —which targets risk factors associated with sexual aggression as well as those associated with violence in general—in ASO.

## Introduction

According to police data, adolescents are responsible for about 21% of all sexual offenses committed in Canada each year (Rotenberg and Cotter, 2018) and a third of crimes against minors in North America are committed by adolescents (Finkelhor et al., 2009; Cotter and Beaupré, 2014). However, more recent data from The National Survey of Children's Exposure to Violence indicate that the majority (over 70%) of sexual abuse and assaults against minors are perpetrated by adolescents (Gewirtz-Meydan and Finkelhor, 2020). It is now recognized, that, contrary to popular belief, adolescents who have sexually offended (ASO) constitute a distinct clientele from adult sex offenders and that few of them will sexually reoffend in adulthood (Lussier and Blokland, 2014).

### Recidivism

The last 20 years have seen increased research into sexual recidivism by adolescents who have sexually offended (ASO). The three most recent meta-analyses of sexual recidivism by adolescents have reported mean rates of 5% (Caldwell, 2016; *N* = 106 studies), 7% (Caldwell, 2010; *N* = 63 studies) and 12% (McCann and Lussier, 2008; *N* = 18 studies); the mean follow-up period for all these was ~5 years. The vast majority of these studies were conducted using adjudicated samples and official data on recidivism. As a result, recidivism rates are possibly underestimated.

The identification of predictors of sexual recidivism is a critically important issue, since a single recidivist may be responsible for a great many victims. Unfortunately, there is still little consensus on reliable predictors of sexual recidivism, especially because of low base rates reported. McCann and Lussier's (2008) meta-analysis identified variables associated with an increased risk of sexual recidivism by ASO: a criminal record for either sexual or non-sexual offending, specific victim characteristics (male, stranger, child/adult but not peer), and evidence of sexual deviance and antisociality. However, these results must be interpreted with caution, given the low effect sizes, and the heterogeneity of the definitions of variables in the studies included. Nevertheless, these results are consistent in large part with those of Worling and Långström's (2006) systematic review of the literature on sexual recidivism. Those authors identified a total of six empirically supported risk factors for sexual recidivism—that is, factors that were statistically associated with sexual recidivism in at least two empirical studies and whose relevance has not been contested by the scientific community: (1) a criminal record for sexual aggression; (2) deviant sexual interests; (3) assault of a stranger; (4) assault of more than one victim; (5) social isolation; and (6) dropping out of specialized treatment. It should be noted that the last two factors were not studied in McCann and Lussier's (2008) meta-analysis.

### Typologies of ASO

Victim age is the most frequently encountered classification criterion in the literature, but has produced inconsistent results. The relationship between recidivism and victim age is unclear. On the one hand, ASO of peers/adults have been reported to exhibit significantly higher rates of sexual (Nisbet et al., 2004; Parks and Bard, 2006), violent non-sexual (Krause et al., 2020) non-sexual (Nisbet et al., 2004), and general (Vandiver, 2006; Fanniff and Kolko, 2012) recidivism. On the other, they have also been reported to exhibit significantly *lower* rates of sexual recidivism (Kemper and Kistner, 2007), as well as *comparable* rates of sexual (Krause et al., 2020), violent (Aebi et al., 2012), non-sexual (Kemper and Kistner, 2007), and general (Hagan et al., 2001; Parks and Bard, 2006; Aebi et al., 2012; Krause et al., 2020) recidivism. In addition, ASO against peers/adults have been reported to be more likely than ASO against children to drop out of treatment (Parks and Bard, 2006). From a theoretical perspective, it has been argued that ASOs against peers/adults are more generalist offenders than specialists, and that sexual offending is one manifestation of a general antisocial orientation (Seto and Lalumière, 2010; Leroux et al., 2016). This hypothesis could explain higher rates of recidivism and poor adherence in treatment (Olver et al., 2011).

Few studies have analyzed mixed ASO (i.e., ASO who had assaulted both children and peers/adults) as a distinct subgroup, despite the empirical evidence in favor of such an analytical strategy (see Kemper and Kistner, 2007; Joyal et al., 2016; Leroux et al., 2016). Mixed ASO are the subgroup of ASO with the greatest treatment needs (Leroux et al., 2016) and are the most likely to drop out of treatment (Kemper and Kistner, 2007).

Victim relationship has not been widely used for classification purposes in ASO, in contrast to adults who have sexually offended (Tidefors et al., 2010). In general, sexual recidivism rates have been reported to be lower in intrafamilial adult sexual aggressors than in extra familial ones (Hanson and Bussière, 1998); however, the reverse has been reported for incestuous young adults (18–24 years), (Hanson, 2002). Among ASO, assault of a stranger victim is generally associated with a higher risk of sexual recidivism (Worling and Långström, 2006; McCann and Lussier, 2008). A recent meta-analysis comparing extra familial and intrafamilial ASOs found higher scores on antisociality indicators among the first group, suggesting a more general pattern of criminality (generalist theory; Martijn et al., 2020). The only reported study to analyses the effect of treatment of ASO on recidivism as a function of victim relationship was that of Latzman et al. (2011; *N* = 166), who used the *Estimate of Risk of Adolescent Sexual Offense Recidivism* (ERASOR; Worling and Curwen, 2001) and the *Youth Level of Service Case Management Inventory* (YLS-CMI; Hoge et al., 2002) to measure the risk of recidivism in intrafamilial and extra familial ASO. Intrafamilial and extra familial ASO did not significantly differ in their global or subscale scores (including the score on the ERASOR treatment subscale) on these two instruments. However, the real rates of recidivism in these two groups were not measured, and mixed ASO were included in the intrafamilial subgroup, rather than considered a distinct group. Finally, the study included only ASO who had received treatment; there was no comparison group (untreated ASO).

### Effectiveness of Treatment, and Recidivism

Current research data suggests that cognitive-behavioral and multi-systemic approaches are more effective in reducing recidivism for ASOs (Walker et al., 2004; Fanniff and Becker, 2006; Kim et al., 2016; Ter Beek et al., 2018). The cognitive-behavioral therapeutic approach (CBT) is more widely used, and is considered to be a first-line treatment, whether in groups or individually (Bereiter and Mullen, 2012; Kim et al., 2016; ATSA, 2017). CBT aims to teach adolescents to identify and modify their thoughts and feelings which support or precipitate abusive sexual behavior (Kim et al., 2016). To this end, CBT develops emotional and behavioral self-regulation skills, in addition to social skills (Rich, 2011). Intervention involves work on accountability for abusive behaviors, the development of empathy, cognitive restructuring, exploring one's own sexual victimization, if applicable, and reducing deviant sexual interests (Center for Sex Offender Management, 2006). Generally, CBT includes relapse prevention strategies, which aim to shed light on the adolescent cycle of abuse, identification of high-risk situations, and the development of internal and external control strategies that reduce risk recidivism (Rich, 2011). This allows the learning and consolidation of new and more adapted behaviors (Sion and Blondeau, 2012). Family involvement and support in the therapeutic process is crucial and can help reduce the risk of recidivism and promote better general functioning (Worling and Curwen, 2000; ATSA, 2017). According to the *Risk-Need-Receptivity* (RNR) model, the intensity of services should be tailored to the level of risk of recidivism and criminogenic needs (Bonta and Andrews, 2007).

Caldwell's (2007) meta-analysis revealed that sexual recidivism by ASO has significantly decreased over the last 15 years, falling from a mean rate of 10.3% in the studies published between 1980 and 1995 to 2.75% in the studies published between 2000 and 2015. In Caldwell's view, at least some of this significant decrease is due to the effectiveness of specialized treatment programs and increased accessibility to these programs. Indeed, the great majority of meta-analyses on this topic have found that ASO who have received specialized treatment of any kind exhibit lower sexual recidivism rates than do those who have not received any treatment (Walker et al., 2004; Reitzel and Carbonell, 2006; Hanson et al., 2009; Schmucker and Lösel, 2015; Ter Beek et al., 2018). For example, in Reitzel and Carbonell's (2006) meta-analysis of nine studies (*N* = 2,986), the mean sexual recidivism rate after a mean follow-up period of 59 months was 7.37% in ASO who had received specialized treatment (*n* = 1,655), and 18.93% in ASO who had received no treatment (*n* = 1,331). While the effect size varied widely between the studies, the results nevertheless suggest that specialized treatment is effective in reducing sexual recidivism. Walker et al.'s (2004) meta-analysis of 10 studies (*N* = 644 ASO) arrived at a similar conclusion. Kim et al.'s (2016) conducted a systematic review that included five meta-analyses published since 2002 (including the two previously described). They found that specialized treatment of any type lowered general recidivism rates (i.e., reoffending of any type) by a mean of 24% compared to no treatment, and was more effective in ASO than in adult sexual offenders. However, sexual recidivism was not specifically analyzed.

The authors of the most recent meta-analysis on this question also concluded that specialized treatment was effective in reducing the rate of general, but not sexual, recidivism in ASO (Kettrey and Lipsey, 2018). Moreover, the authors call in to question the reliability of the conclusions of previous meta-analyses, the majority of which included studies with non-experimental designs. They also note that their own meta-analysis' statistical power—and by extension, ability to detect more modest effects on sexual recidivism—was limited by the small number of studies they analyzed that had used, an experimental design (*N* = 7), and the modest effect sizes.

### Treatment Dropout and Recidivism

While the vast majority of studies of the effectiveness of treatment programs have compared ASO who received treatment to those who had not (either no treatment at all or unspecialized treatment), Edwards et al.'s (2005) compared ASO who had completed specialized treatment programs to those who had dropped out. In their sample (*N* = 49), general, violent, and sexual recidivism rates after a mean follow-up period of 3 years were higher in ASO who had dropped out of treatment than in those who had completed treatment: in fact, none of the ASO who completed their treatment programs committed a sexual offense in the follow-up period. These results are interesting, as the significant differences observed between ASO who completed treatment and those who dropped out suggest that it is important to study these two subgroups as distinct entities, something few studies have done to date. Unfortunately, it is difficult to generalize from these results, given the study's small sample size, the relatively short follow-up period, and the low recidivism rates.

### Weaknesses of Studies of the Association Between Treatment and Recidivism

In their review of studies published between 1986 and 2005 on recidivism by ASO, Fortune and Lambie (2006) concluded that the majority of studies were characterized by methodological weaknesses, and that these weaknesses were primarily in three domains: (1) characteristics of the populations studied, and the absence of control groups; (2) definition of recidivism; (3) duration of follow-up period. Their criticisms remain pertinent today, as very few studies have addressed all these issues (Kettrey and Lipsey, 2018).

First, Fortune and Lambie (2006) observed that most studies do not use appropriate control groups (ASO who had not received specialized treatment or who had not completed treatment). In addition, they acknowledge that obvious ethical considerations render randomization of subjects to groups (specialized treatment vs. no treatment or unspecialized treatment) for the purposes of comparison difficult, if not impossible. They therefore suggested that a more useful approach to evaluating the effectiveness of treatment in reducing recidivism by ASO would be to compare groups who had received treatment to those that had not received treatment or had not completed treatment (p. 1,090). They also note the importance of taking into consideration the criteria that govern the (non-random) allocation of participants to treatment groups. More specifically, they recommend taking into account not only the usual variables (e.g., age, gender, socio-economic status) but also variables that may influence selection of candidates for treatment or candidates' ability to complete treatment (e.g., comorbid psychiatric conditions, history of sexual victimization). Finally, they emphasize the importance of ensuring samples are large enough to ensure representability and the construction of groups with comparable personal, familial, and offending characteristics.

Second, given the low base rates of sexual recidivism and the higher rates of non-sexual and general recidivism, Fortune and Lambie recommend measuring the effectiveness of treatment in terms of all three recidivism rates. In addition, they suggest using several sources of information on recidivism (official and non-official); should this be impossible, they suggest using an official source that best reflects real offending behavior (e.g., charges rather convictions, to avoid the effects of plea bargaining). Third, they recommend using follow-up periods that are long enough to allow evaluation of the effects of treatment on short-, medium-, and long-term recidivism.

### Current Study

The current study was designed to avoid the three major weaknesses outlined above. First, it uses a large (*N* = 351) sample of ASO, all of whom had undergone a multidisciplinary (e.g., psychology, psychiatry, criminology) evaluation that was adapted to sexual offenders and intended to direct them to specialized treatment. Second, the study comprises three groups: (1) ASO who had completed specialized treatment (*completed treatment*); (2) ASO who received no treatment (*no treatment*); and (3) ASO who had commenced treatment but dropped out before the treatment was completed (*dropped out*). The sexual, violent (including sexual), and general recidivism rates of the *completed treatment* group (experimental group) was compared to those of the other two (control) groups. Third, the mean follow-up period was almost 8 years (range = 2–13 years), long enough to allow evaluation of recidivism. Fourth, data on recidivism was collected from multiple official sources, namely Youth Court (recidivism in adolescence), and municipal, provincial, and federal courts (recidivism in adulthood). In keeping with the recommendation of Fortune and Lambie (2006) and Caldwell (2007), recidivism was defined in terms of charges laid, to avoid the effect of plea bargaining. Finally, the current study not only takes into consideration the principal methodological recommendations of Fortune and Lambie (2006), but also analyses recidivism and participation in treatment in terms of two types of typologies, victim age (children, peers or adults, mixed) and victim relationship (intrafamilial, extra familial, intra/extra familial). The validity of those subtypes has been supported by some studies (Kemper and Kistner, 2007; Latzman et al., 2011; Joyal et al., 2016; Leroux et al., 2016; Krause et al., 2020; Martijn et al., 2020). This is the first published study aiming to measure the effect of this unique treatment offered in Quebec (Canada) on reducing the recidivism rates of ASOs.

### Objectives and Hypotheses

The primary objective of this study was to evaluate the effectiveness of a specialized treatment program designed to reduce recidivism by ASO. A secondary objective was to determine whether completion of treatment and recidivism are associated with victim age or victim relationship.

The study had four hypotheses based on previous empirical research and theoretical considerations:

1. ASO who complete treatment present significantly different rates of sexual, violent, and general recidivism, compared with ASO who do not receive treatment or who drop out of treatment
2. Completion of treatment reduces the probability of sexual, violent, and general recidivism
3. ASO against children are more likely to complete treatment than are ASO of peers/adults or mixed ASO, and have lower violent and general recidivism rates
4. Intrafamilial ASO are more likely to complete treatment than extra familial ASO, and have lower sexual, violent, and general recidivism rates.

## Method

### Treatment Program

The Institute Philippe-Pinel de Montréal (Quebec, Canada) offers a specialized outpatient evaluation and treatment program for adolescents (12–18 years old) who have committed sexual offenses. Referrals can be made by Youth Court case workers and by professionals in the health and social services network, including Quebec Youth Centers. Adolescents are referred from across the province, but the largest group comes from the Greater Montreal Area. Adolescents referred to the clinic undergo an initial evaluation, intended to establish their sexual issues, and treatment needs, by a multidisciplinary team of professionals (psychiatrist, psychologist, criminologist). If enrolment in a specialized treatment program is indicated and the adolescent exhibits at least minimal motivation to enter treatment or acknowledgment (acknowledgment of some facts or of the existence of sexual problems), he or she is admitted to a treatment program.

In all cases, the approach is cognitive-behavioral therapy targeting relapse prevention. The treatment program targets three main objectives: (1) fostering a better understanding of the offending process, i.e., the sequence of factors that predispose to, and trigger, sexual aggression; (2) developing control and avoidance strategies that prevent recidivism; and (3) favoring a return to healthy psychosexual development, particularly by addressing factors that are not specific to sexual aggression (e.g., self-control, emotional regulation, relational and social skills, prosocial cognitions, self-esteem, empathy). This group therapy is delivered weekly for ~26 weeks (90 minutes per week) by two therapists of the multidisciplinary team. The therapy groups are closed, and are composed of six to nine adolescents who have no developmental impairment; to the extent possible, the groups are stratified by age (12–15 years and 15–18 years). The program was implemented in the 1990s and has been periodically revised, based on ATSA guidelines and evidence from the scientific literature. Three of the program's founding therapists, including a psychiatrist, still conduct therapy and act as trainers and supervisors for the program. Themes are addressed in the form of theoretical or practical exercises (e.g., family history, significant life events, pre-abuse, abuse and post-abuse period, consequences of sexual assault, letter to the victim, healthy sexual development and intimacy). An unpublished treatment manual is available to therapists.

Progress in therapy is assessed at mid-therapy and at the end, in case management meetings with adolescents, parents and case workers. Adolescents who have not reached the treatment goals, who do not satisfy the basic criteria for group therapy (e.g., developmental impairment, commission of a non-contact sexual offense), or for whom group therapy is contraindicated are referred for individual cognitive-behavioral therapy, the duration of which depends on their needs. Individual therapy is delivered by a psychologist of the team, with the same objectives as the group therapy. In both the group and individual treatment programs, there are regular meetings with parents and case workers in contact with the adolescent, in order to foster teamwork and ensure that the adolescent can develop in an environment that is safe for him and others. The vast majority of adolescents also benefited from psychosocial services; some also benefited from other clinical services, such as rehabilitation and family interventions.

### Participants

The initial sample was composed of 351 male ASO who were evaluated at the Institute Philippe-Pinel outpatient clinic between 1992 and 2002. The sample has been previously described (Carpentier and Proulx, 2011). Following exclusion of 24 initial participants due to the destruction or unavailability of files, the final sample comprised 327 participants. The mean age of the participants at the time of evaluation was 15.8 years (*SD* = 1.9). Almost all (95.1%) of the subjects had been born in Canada; 1.5% (*n* = 5) had been born in Africa, 2.3% (*n* = 8) had been born in Central America, and information on three participants was missing. At the time of their initial evaluation, 24.1% of the participants were facing a criminal charge under the Young Offenders Act, 49.6% were in care or were being followed under the Youth Protection Act, and 19.9% were followed under both acts^1^. Only 5.5% (*n* = 8) of the participants were not facing charges or warrants. During initial evaluation, it was estimated that 208 adolescents had committed sexual assaults against children only (victim younger than 12 years and aggressor at least 3 years older) and that 88 had sexually assaulted only peers or adults (same age group as aggressor, or an adult, i.e., older than 17 years). The rest of the sample (*n* = 31) was composed of adolescents who had assaulted victims in both age categories (mixed aggressors). The victim relationship was intrafamilial only (biological sibling) in 30.0% of cases (*n* = 98), extra familial (e.g., sibling in a blended family, cousin, classmate, stranger) in 57.5% (*n* = 188), both intrafamilial and extra familial in 11.9% (*n* = 39, and unknown in 0.6% (*n* = 2). For the index sexual offense (the most recent offense at the time of evaluation), the mean age of the victim was 9 years (range = a few months to 41 years, *SD* = 5.42); age was unknown in five cases. The majority of the victims (65.4%) were female. The mean number of known victims per participant was 2.29 (range = 1–16, *SD* = 1.94), yielding a total of 748 victims for the entire sample.

### Procedure

#### Data Collection

Data collection proceeded in three phases. In the first phase, retrospective data was collected from all available sources: multidisciplinary evaluation produced by the Institute Philippe-Pinel outpatient clinic, psychological evaluations, summary reports of social services, presentencing reports, police reports, and victim statements. More than 800 variables on individual, familial, social, and offending factors were coded. The lead researcher and two research assistants participated in this phase of data collection. To determine the reliability of coding, agreement between the principal researcher and one research assistant, was analyzed, using a sample of 20 subjects. The weighted kappa was 0.95 (0.71–1.00), indicating almost perfect agreement. The scoring of the other research assistant was supervised and validated by the first two coders. Definitions of variables included in this study are described in the Appendix (see Supplementary Material).

#### Recidivism

In the second phase, recidivism was evaluated on the basis of official juvenile and adult criminal records. Data up until 2005 was collected from the archives of Youth Court and municipal, provincial, and federal adult courts. Recidivism was defined as any new charge (excluding parole violations and breaches of conditions) during the follow-up period following the participant's initial multidisciplinary evaluation; evaluating recidivism in terms of charges rather than convictions avoids the negative effects of plea bargaining (Caldwell, 2007). In keeping with the recommendation of Fortune and Lambie (2006), three types of recidivism were analyzed:

1. General recidivism, defined as any criminal charge in the follow-up period
2. Violent recidivism, defined as a charge related to a crime against persons (including a sexual crime) in the follow-up period
3. Sexual recidivism, defined as a charge related to a contact or non-contact sexual offense during the follow-up period

The mean follow-up period was 94.0 months (range = 21–162 months, *SD* = 32.2). The mean age of the participants at the end of the follow-up period was 23.9 years (range = 17–32 years). By the end of the follow-up period, 43.7% (*n* = 143) of the sample had been charged with a new criminal offense of some kind. Among the recidivists, 28.1% (*n* = 92) of the total sample had been charged with a new violent offense (including sexual offenses) and 10.1% (*n* = 33) had been charged with a new sexual offense.

#### Treatment

Data on treatment offered following the initial evaluation was collected retrospectively from the archives of the Institute Philippe-Pinel de Montréal (case notes, treatment summaries, closure memos, etc.). The significant lapse of time between the first phase of data collection and the collection of data on treatment partially explains the fact that 24 of the 351 files initially consulted were not available or had been destroyed. The lead researcher and two research assistants used a checklist to collect data on treatment-related variables, especially participation (or non-participation) in treatment, treatment completed, etc. The first 20 files were coded independently by the three coders. In the great majority of cases, no discrepancies were observed, no doubt reflecting the factual nature of the data collected. The few discrepancies in scoring were analyzed until a consensus was reached and the coding was revised. The remaining files were coded in keeping with established practices.

Following initial evaluation, a total of 150 (45.9% of the sample) participants commenced specialized treatment in the Institute Phillipe-Pinel de Montréal's outpatient clinic, on the basis of recommendations of the initial multidisciplinary evaluation; 177 participants were not admitted to treatment, primarily because they failed to acknowledge the existence of a sexual issue (*n* = 108) and/or lacked motivation to undertake treatment (*n* = 84). Other reasons included the existence of other, higher priority, issues (*n* = 10), such as psychopathologies, aggressivity and trauma symptoms, or the fact that no sexual issue had been identified (*n* = 19).

Some of them were also referred elsewhere for geographical reasons (*n* = 19). Among the 150 admitted, only 62 (41.3%) successfully completed treatment, and 84 (56.0%) dropped out or were expelled. The vast majority of adolescents who were expelled had accumulated too many absences (a kind of abandonment of the therapy). A few others had broken the rules (e.g., inappropriate behavior during therapy, lack of respect), which is usually a sign of lack of motivation and commitment to therapy. Before expelling an adolescent from therapy, a meeting was carried out with him, his parents and his caseworker. Generally, therapy was stopped with the agreement of all. Information on treatment completion was missing for four. Consequently, analyses were conducted on a total of 323 subjects.

## Analyses

Statistical analyses were performed with IBM SPSS, version 27.0. The similarity of 20 dichotomous (yes/no) and four continuous variables in the three groups (*completed treatment n* = 62; *dropped out n* = 84; *no treatment n* = 177) was analyzed using bivariate statistics (chi square and Kruskal-Wallis tests). Effect size was measured by Cramer's V, with a V of 0–0.10 considered to indicate no effect, 0.11–0.20 a weak effect, 0.21–0.3 a moderate effect, and >0.30 a large effect (Fox, 2009). The threshold for clinical relevance was set at 0.21 (moderate effect size) and the level of statistical significance for *p*-values was set at 0.05. Subsequently, additional chi square tests were performed, in order to detect significant differences in sexual, violent, and general recidivism in the three groups (*completed treatment, dropped out, no treatment*). In order to verify Hypothesis 1 (significant differences between the *completed treatment* group and the rest of the sample), Kaplan-Meier survival analyses were performed. This type of analysis takes into account the time elapsed until a new offense and the total follow-up period for participants who did not commit a new offense (censored data). In order to verify Hypothesis 2 (completion of treatment reduces the probability of sexual, violent, or general recidivism) Cox regression analyses were performed. Cox regression analyses are used to assess the effect of risk factors (considered simultaneously) on survival time. The hazard ratio [Exp (B)], give estimation of the effect size of each risk factor (covariate) when the other covariates are controlled. In order to create distinct predictive models for each type of recidivism, three separate analyses were conducted, using a Wald stepwise descending method. Descriptive variables exhibiting a moderate effect size and an acceptable level of significance (*p* < 0.05), (Table 1) were entered into the model's first block in order to control for their possible effects. Finally, supplemental analyses (logistic and Cox regressions) were performed to test hypotheses 3 and 4.

**Table 1:** Mean (SD) and distribution of variables in the three groups (treatment completed, dropped out, no treatment).

	**Treatment completed *N* = 62**	**Dropped out *N* = 84**	**No treatment *N* = 177**	***H***	***p***	
Age	15.6 (1.83)	15.6 (1.72)	15.9 (1.78)	2.38	0.305	
Follow-up period (months)	99.82 (34.10)	96.83 (32.23)	90.16 (31.47)	5.01	0.082	
Age at first sexual assault	13.2 (2.06)	12.4 (2.65)	13.1 (2.08)	4.45	0.108	
Total number of known victims	2.05 (1.75)	2.83 (2.49)	2.10 (1.64)	8.90	**0.012**	
	%	%	%	χ^2^	*p*	ES
Parents separated	60.0	79.8	77.5	8.71	0.013	0.166
Out-of-home placement	59.7	84.5	63.8	13.99	0.001	**0.208**
Neuropsychiatric history						
Conduct disorder	29.0	29.8	31.6	0.19	0.910	0.024
ADHD	29.0	29.8	24.9	0.87	0.648	0.052
Low IQ	11.3	20.2	15.3	2.32	0.328	0.083
History of victimization						
Sexual violence	40.3	38.1	29.9	3.03	0.220	0.097
Physical violence	29.0	42.7	35.3	2.94	0.230	0.096
Parental neglect	48.4	69.0	55.4	7.02	0.030	0.147
Social skills						
Isolation/social rejection	75.8	81.0	64.4	8.45	0.015	0.162
Atypical sexual interests						
Deviant sexual fantasies	61.3	59.5	41.2	11.64	0.003	0.190
Male victim	40.3	61.9	36.2	15.70	0.000	**0.220**
Stranger victim	4.8	8.3	5.6	0.95	0.622	0.054
Deviant sexual behaviors	33.9	39.3	36.7	0.45	0.798	0.037
Anti-sociality/delinquency						
Criminal record	6.5	19.0	19.8	6.09	0.047	0.137
Delinquent peers	13.6	25.3	29.6	5.91	0.052	0.140
Alcohol/drug consumption	27.5	40.6	47.2	5.84	0.054	0.156
Early-onset aggressivity	40.3	61.9	54.2	6.75	0.034	0.145
Physical violence to peers	50.0	59.5	57.6	1.47	0.480	0.067
Victim age				11.84	0.019	0.135
Child (*n* = 208)	21.5	29.8	48.8			
Peer/adult (*n* = 88)	16.1	14.9	69.0			
Mixed (*n* = 31)	12.9	32.3	54.8			
Victim relationship				4.86	0.302	0.087
Intrafamilial (*n* = 98)	18.6	23.7	57.5			
Extra familial (*n* = 188)	19.4	24.7	55.9			
Intra/extra familial (*n* = 39)	21.1	39.5	39.5			

*All these variables were evaluated at the time of initial evaluation. H, Kruskal-Wallis; χ^2^, chi square; ES, Effect size*.

*Bold: significant at p < 0.005 and effect size > 0.20*.

## Results

### Comparative Analyses

In order to verify the homogeneity of the three groups, the groups were compared on several variables of interest (Table 1). No significant difference was observed for three of the four continuous variables: age of participants [*H(2)* = 2.38, *p* = 0.305], duration of follow-up (*H* = 5.01, *p* = 0.082) and age at first sexual assault [*H(2)* = 4.45, *p* = 0.108]. However, the mean number of known victims at the time of initial evaluation was slightly higher in the *dropped out* group than in the other two groups [2.8 vs. 2.1 and 2.05, *H(2)* = 8.90, *p* < 0.05]. The groups were comparable for almost all dichotomous variables. The only significant differences observed were for placement [χ^2^(2) = 13.99, *p* = 0.001, *ES* = 0.208] and male victim [χ^2^(2) = 15.70, *p* < 0.001, *ES* = 0.220], and the effect sizes for these variables were moderate. ASO in the *dropped out* group were significantly more likely than ASO in the other two groups to have been in out-of-home care (placement) at the time of initial evaluation and to have assaulted at least one male. While significant differences were observed for victim age, the effect size (0.14) was below the threshold of clinical significance. No significant difference was observed between groups for victim relationship (intrafamilial, extra familial, intra/extra familial).

The recidivism rates of each of the three ASO groups are presented in Table 2. The mean follow-up period was 7.8 years. The *completed treatment* group exhibited a sexual recidivism rate similar to the *no treatment* group, and lower violent and general recidivism rates than the other two groups. However, no significant intergroup differences were found.

**Table 2:** Sexual, violent, and general recidivism for the three groups (treatment completed, dropped out, no treatment).

**Variable**	**Treatment completed (*****n*** **=** **62)**		**Dropped out (*****n*** **=** **84)**		**No treatment (*****n*** **=** **177)**		***X^**2**^***	***p***	**ES**	**95% CI**
	***n***	**%**	***n***	**%**	***n***	**%**				
Type of recidivism										
Sexual	5	8.1	13	15.5	14	7.9	3.946	0.139	0.111	[0.048–0.236]
Violent (including sexual)	10	16.1	25	29.8	5	31.1	5.305	0.070	0.128	[0.048–0.236]
General	24	38.7	39	46.4	78	44.1	0.891	0.640	0.053	[0.015–0.170]

*χ^2^, chi square; ES, Effect size; 95% CI, 95% confidence interval. Mean follow-up period, 94 months (range = 21–162 months, SD = 32.2)*.

**Figure 1:**
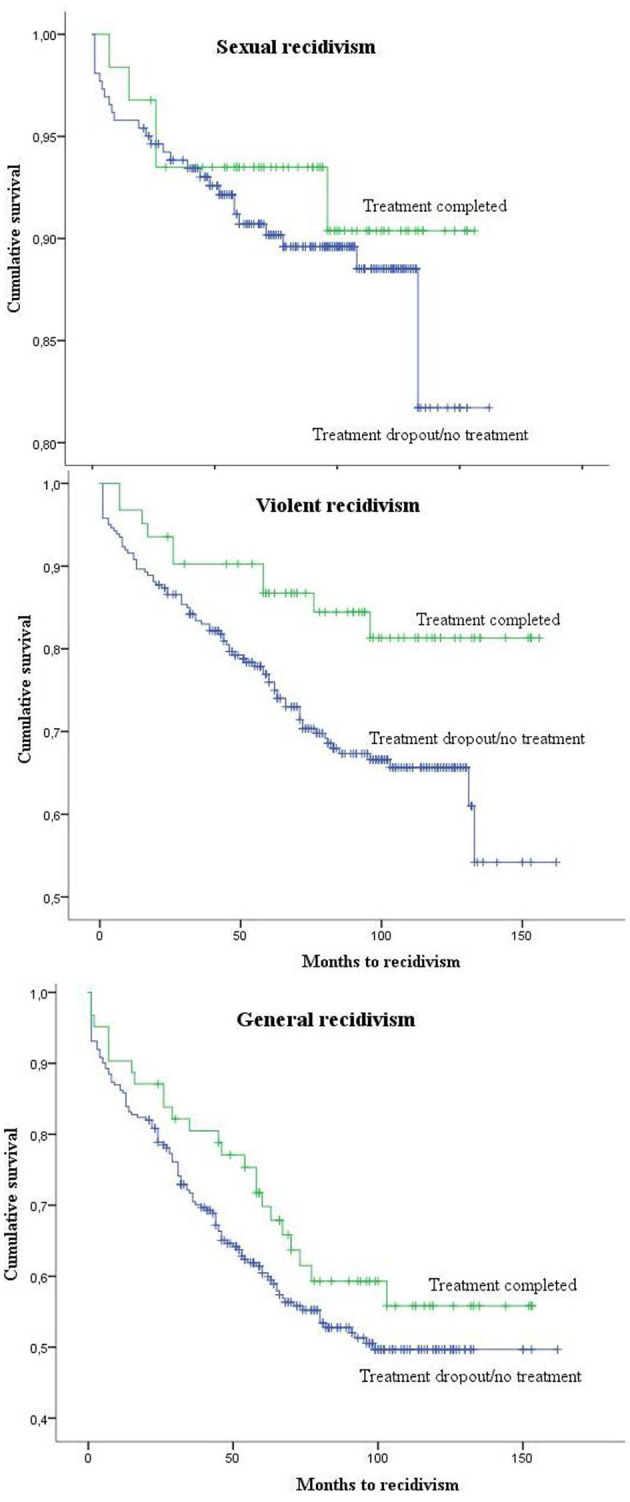
Kaplan-Meier survival curve estimates for sexual, violent (including sexual) and general recidivism by the treatment completed group and the dropped out/no treatment group.

As suggested by Worling and Curwen (2000), Kaplan-Meier survival analyses (Log rank) were performed, in order to take into account the period of time elapsed between initial evaluation and recidivism. The three groups (*completed, dropped out, no treatment*) were compared on sexual, violent, and general recidivism. No significant difference was found between the groups for sexual [χ^2^ (2, *N* = 323) = 3.80, *p* = 0.150] and general recidivism [χ^2^ (2, *N* = 323) = 1.59, *p* = 0.451], but a significant difference was found for violent recidivism [χ^2^ (2, *N* = 323) = 6.09, *p* = 0.048]. The rate of violent recidivism in the *treatment* group was almost half that in the *dropped out* group and the *no treatment* group (16.1 vs. 29.8% and 31.1%). The two comparison groups (*dropped out* and *no treatment*) were merged to form a single group (*n* = 261), which was then compared to the *completed treatment* group (*n* = 62). Again, violent recidivism differed significantly in the two groups, with the *completed treatment* group exhibiting a violent recidivism rate almost half that of the comparison group [16.1 vs. 30.7%, χ^2^ (1, *N* = 323) = 5.98, *p* = 0.014]. The rates of sexual and general recidivism were lower in the *completed treatment* group (8.1 vs. 10.3%, and 38.7 vs. 44.8%), but not significantly so (Figure 1).

### Multivariate Analyses

To determine whether completion of the specialized treatment reduces the probability of reoffending, Cox regression analyses were performed, with control for placement, number of known victims at the time of initial evaluation, and male victim, all of which exhibited a moderate effect size and an acceptable level of significance (*p* < 0.05). The variables were entered into two blocks—the control variables in block 1 and the “treatment completed” variable in block 2—using Wald's stepwise descending method (Table 3). The results indicate that completion of the specialized treatment program did not significantly decrease the probability of sexual or general reoffending, but did reduce the probability of violent (including sexual) reoffending by almost half. The final model is significant [χ^2^ (2, *N* = 323) = 8.883, *p* = 0.012] and includes the “history of placement” control variable.

**Table 3:** Final cox regression predicting violent recidivism (including sexually).

**Variable**	**B**	**SE**	**Wald**	**df**	***p***	**Exp (B)**	**95% CI for Exp(B)**	
							**Min**	**Max**
Block 1								
Step 1								
Placement	0.477	0.252	3.571	1	0.059	1.611	0.982	2.643
Male victim	0.137	0.222	0.383	1	0.536	1.147	0.743	1.772
Number of known victims	−0.020	0.055	0.133	1	0.715	0.980	0.879	1.092
Step 2								
Placement	0.465	0.250	3.443	1	0.064	1.591	0.974	2.600
Male victim	0.113	0.212	0.283	1	0.595	1.120	0.739	1.697
Step 3								
Placement	0.475	0.250	3.624	1	0.057	1.608	0.986	2.623
Block 2								
Placement	0.429	0.250	2.944	1	0.086	1.536	0.941	2.507
Treatment completion	−0.760	0.337	5.077	1	0.024	0.467	0.241	0.906

*Wald's stepwise descending method was used*.

Supplemental analyses were performed (Hypothesis 3 and 4) to determine whether victim age and relationship were associated with: (1) the completion of treatment (logistic regressions); and (2) sexual, violent, or general recidivism rates (Cox regressions). Neither of the two logistic regression models was significant: neither victim age (*p* = 0.163) nor victim relationship (*p* = 0.751) had any effect on the completion of treatment. However, while victim age did not have a significant effect on sexual (*p* = 0.767) or general (*p* = 0.159) recidivism, it did influence the probability of violent recidivism [χ^2^ (2, *N* = 327) = 6.247, *p* = 0.044]: ASO who had sexually assaulted peers/adults were almost twice as likely to violently (including sexually) reoffend [Exp (B) = 1.754, *p* = 0.014]. Victim relationship had no significant effect on any form of recidivism (sexual: *p* = 0.287; violent: *p* = 0.421; general: *p* = 0.724).

## Discussion

The main objective of this study was to evaluate the effectiveness of a specialized treatment program in reducing recidivism by ASO. Several measures were taken in order to limit the methodological biases generally associated with this type of study: (1) the sample was quite large in the context of adolescents who sexually offend (*N* = 327); (2) only male ASO who had committed at least one contact sexual assault were recruited; (3) the study design included two control groups (*dropped out* and *no treatment*); (4) to limit selection bias and control for variables with significant differences, the experimental (*completed treatment*) group was compared to the control groups over 24 pretreatment variables; (5) three types of recidivism (sexual, violent, general) were analyzed, in both adolescence and adulthood, and the criterion for recidivism was criminal charges, rather than convictions; (6) the follow-up period was up to 13 years following initial evaluation (*M* = 7.8 years, *SD* = 2.7).

The results of this study provide evidence to support the effectiveness of a specialized, cognitive-behavioral, treatment program in reducing violent (including sexual) recidivism in ASO over a mean follow-up period of almost 8 years. Other studies have reported the effectiveness of specialized treatment programs in reducing sexual, non-sexual, or general recidivism by ASO (see Walker et al., 2004; Reitzel and Carbonell, 2006; Borduin et al., 2009, and the meta-analyses by Hanson et al., 2009; Kim et al., 2016; Kettrey and Lipsey, 2018). The effectiveness of the treatment program evaluated here in reducing violent recidivism of all kinds may be due to the fact that it targeted not only factors specific to sexual aggression (e.g., attitudes supporting sexual offenses) but also non-specific factors associated with other types of offending (e.g., emotional regulation, prosocial cognitions, relational skills). Moreover, the fact that ASO exhibited higher rates of non-sexual and general recidivism than of sexual recidivism highlights the necessity of offering treatment that targets not only sexual issues but also factors associated with violent and general offending (McCann and Lussier, 2008; Worling et al., 2010; Pullman and Seto, 2012). The results did not reveal significant differences between the rates of sexual and general recidivism in the *completed treatment* and comparison groups, but it should be noted that the low base rates of sexual recidivism (10.1% for the entire sample) compromised statistical power (Barbaree, 1997; Quinsey et al., 2006; Kemper and Kistner, 2007). In addition, the comparability (8%) of sexual recidivism rates in the *completed treatment* and *no treatment* groups may be due to the fact that many ASO in the *no treatment* group did not exhibit sexual issues requiring specialized treatment during the initial evaluation, or that some may have received specialized treatment in another region. Unfortunately, neither of these items of information was available in the files.

Although some authors (Becker and Johnson, 2001; Walker et al., 2004) have suggested that treatment effectiveness should be evaluated for each type of ASO, this has rarely been done. In the present study, treatment effectiveness and recidivism were evaluated in terms of two variables frequently used in studies of ASO: victim age and victim relationship. As suggested by Leroux et al. (2016), mixed ASO, i.e., ASO who had assaulted both children and peers/adults, were considered a distinct subgroup. The bivariate analyses indicate that mixed ASO had the lowest rate of treatment completion of the three groups. Although the effect size was low, perhaps because of the limited number of mixed ASO, these results are consistent with the results of other studies which have reported mixed ASO to have the highest treatment non-completion rates (Parks and Bard, 2006; Kemper and Kistner, 2007; Lillard et al., 2020), and suggest that mixed ASO have more complex issues and greater treatment needs (Leroux et al., 2016; Lillard et al., 2020).

In Leroux et al.'s (2016) study, mixed ASO exhibited the most problematic clinical portrait. They were significantly more likely than peer/adult or child ASO to have a history of sexual victimization, atypical sexual fantasies, and ADHD, and to have been cruel to animals. In addition, like the peer/adult group, they were more likely than child ASO to have criminal or psychiatric records and conduct disorders, and to be sexually experienced. The number and combination of factors related to both general and sexual offending in mixed ASO suggests that their risk and treatment needs are higher than those of the other two groups, and that failure to adapt treatment programs to their specific needs could lead to treatment drop out or failure (cf. the RNR Model; see Andrews and Bonta, 2010; Koehler et al., 2013).

Our complementary analyses (Cox regressions) indicate that victim age influences the probability of recidivism: ASO who had sexually assaulted peers or adults were twice as likely to violently (including sexually) reoffend. Some studies have reported that ASO against peers generally exhibit more antisocial behaviors and factors related to general offending that do ASO against children (Awad and Saunders, 1991; Richardson et al., 1997; Parks and Bard, 2006; Gunby and Woodhams, 2010; Seto and Lalumière, 2010; Aebi et al., 2012; Fanniff and Kolko, 2012; Glowacz and Born, 2013; Zeng et al., 2015; Joyal et al., 2016; Leroux et al., 2016; Krause et al., 2020). However, the results in the literature concerning the relationship between victim age and recidivism are inconsistent. This inconsistency may be due to: (1) the absence of consensus on the definition of ASO types (e.g., age criteria, use of most recent offense vs. all recorded offenses); (2) the (non-)classification of mixed ASO as a distinct group; (3) the duration of follow-up; (4) the size and source of the sample (e.g., courts, specialized treatment centers); (5) the definition of recidivism (charges vs. convictions); and (6) the low base rates of sexual recidivism (which render high statistical power elusive).

In order to address this last limitation, the majority of studies that have investigated the association between typologies and recidivism have focused on non-sexual or general recidivism; few of these have reported significant differences related to victim age. Our results confirm that the three types of ASO (against children, adults/peers, mixed) exhibit comparable sexual and general recidivism rates. Recent studies have also demonstrated that all three types of ASO are more similar than different, especially from developmental, familial, and social perspectives (Aebi et al., 2012; Fanniff and Kolko, 2012). However, ASO against children generally exhibit fewer externalized problems and more internalized ones (e.g., anxiety, depression, low self-esteem) than ASO against peers (Whitaker et al., 2008), as well as a more asocial or sexually deviant, rather than antisocial, profile (Joyal et al., 2016), which may explain their lower propensity to violence, except sexual aggression. Overall, these results and hypotheses require further study.

Contrary to our initial hypotheses, treatment completion and sexual, violent, and general recidivism were not significantly different in intrafamilial ASO than in extra familial and intra/extra familial ASO. This result confirms that ASO differ from adult sexual offenders, in which intrafamilial sexual aggression is associated with a lower risk of sexual recidivism than extra familial aggression (Hanson and Bussière, 1998; Hanson, 2002). However, Hanson (2002) has demonstrated that the association between recidivism and victim type was limited to young adult (18–24 years) sexual offenders, which suggests that these offenders are more similar to ASO than to adult sexual aggressors. The comparison of these results should be interpreted with caution, since the categorizations used by Hanson in both studies differ from those used in this one.

More than half of participants admitted to treatment dropped out or were expelled, which is in line with attrition rates reported in juvenile rehabilitation programs but higher than those reported in cognitive-behavioral sex-offender treatment (Olver et al., 2011). As showed in Table 1, the participants in the dropout group in this study exhibited higher anti-sociality (previous criminal record, delinquent peers, alcohol or drug consumption, early-onset aggressivity, physical violence to peers), compared with treatment completer group, which could explain a part of their treatment attrition. History of antisocial behaviors and antisocial orientation are recognized as strong risk factors for treatment attrition among overall juvenile offender and sex offenders (Edwards et al., 2005; Larochelle et al., 2010; Olver et al., 2011). The dropout group had the highest rate of out-of-family care (placement), in addition to having a high rate of parental neglect. Since it is known that family support increases the completion of treatment and decreases the risk of recidivism (Yoder et al., 2015), it is possible that these factors affected the dropout group, which could explain the poor adherence to treatment and higher rates of recidivism. Unfortunately, because we were unable to measure this variable (family support), we cannot confirm this hypothesis.

High rates of treatment dropout also possibly reflect issues of responsivity in the treatment program. According to this principle, a component of the risk, need, and responsivity model (RNR; Andrews and Bonta, 2006, 2010), more effective treatment programs are cognitive behavioral in orientation (general responsivity) and must be adapted to participants' learning styles, cognitive capabilities, insights, motivations, and cultural and personality factors such as antisocial traits (specific responsivity). Although this program adheres to the principle of general responsiveness, it may be less suitable for participants who are less motivated or resistant to interventions. It is also possible that adolescents who have dropped out were at a higher risk level and needed more intensive treatment than that provided by the outpatient program. Unfortunately, these assumptions could not be verified since the variables related to risk, need and responsiveness were not measured in this study.

## Limitations and Future Directions

Some limitations of this study should be noted. First, the sample was composed solely of male adolescents who had committed a contact sexual offense, and who had been referred to an outpatient clinic for specialized evaluation. These were therefore adolescents who had been charged—and, in most cases, convicted—for a sexual assault. It is possible that participants are not representative of the overall population of adolescents who have sexually offended. Second, data collection was entirely archive-based and retrospective. Consequently, the information available was limited to that contained in each adolescent's file. Third, the length of time spent in specialized treatment, the form of treatment (group or individual), and the application of other measures (e.g., psychosocial and family interventions, legal measures) in conjunction with the treatment were not considered. For example, it is possible that adolescents who completed the specialized treatment program also received more intense or more extended services; unfortunately, the data is insufficient to allow conclusions to be drawn in this regard. Fourth, treatment effectiveness was evaluated solely in terms of recidivism, despite the fact that this treatment also targets cognitive (e.g., prosocial cognitions), emotional (e.g., emotional regulation), social (e.g., social skills), and other factors. Unfortunately, the retrospective design of this archive-based study did not allow evaluation of these changes or of changes related to the social adaption of participants before and after treatment. Finally, this is a non-randomized study. Consequently, results should be interpreted with caution, since they could be attributable to differences between groups that could not be measured.

A study by Fanniff et al. (2017) has demonstrated the scientific and clinical value of analyzing not only recidivism, which should also encompass self-report offenses, but also social adaptation, especially educational and work engagement and quality of interpersonal relationships with peers and adults. Taking all these variables into consideration could result in more complete evaluations of treatment effectiveness, and a better understanding of the complex process of desistance from offending (Maruna, 2004; Serin and Lloyd, 2009).

## Data Availability Statement

The raw data supporting the conclusions of this article will be made available by the authors, without undue reservation.

## Ethics Statement

The studies involving human participants were reviewed and approved by Comité d'éthique de la recherche de l'Université du Québec à Trois-Rivières and Comité d'éthique de la recherche de l'Institut Philippe-Pinel de Montréal. Written informed consent from the participants' legal guardian/next of kin was not required to participate in this study in accordance with the national legislation and the institutional requirements.

## Author Contributions

JC and JP: designed the study, developed the coding form, revised the manuscript and both approved the submitted version. JC collected the data, conducted statistical analyses, and wrote the first draft of the manuscript. All authors contributed to the article.

## Funding

A part of this research was support by a grant from Social Science and Humanities Research Council of Canada.

## Conflict of Interest

The authors completed this study while part-time employed by Institut national de psychiatrie Légale Philippe-Pinel.

## Publisher's Note

All claims expressed in this article are solely those of the authors and do not necessarily represent those of their affiliated organizations, or those of the publisher, the editors and the reviewers. Any product that may be evaluated in this article, or claim that may be made by its manufacturer, is not guaranteed or endorsed by the publisher.

## Footnotes

^1^In Quebec, sexual offences committed by adolescents are governed by two laws: the Youth Protection Act and the Youth Criminal Justice Act (prior to 2003, Young Offenders Act). An ASO may be taken into custody under either of the two laws, depending on precise criteria (for more details, see Alain et al., 2018).
