# Supplementary material for: Recidivism Rates of Treated, Non-Treated and Dropout Adolescent Who Have Sexually Offended: a Non-Randomized Study
Source: Front Psychol. 2021 Oct 13;12:757242. doi: 10.3389/fpsyg.2021.757242 (PMC8548636; doi:10.3389/fpsyg.2021.757242)
Supplement: Supplementary file 1 [file Data_Sheet_1.docx]

Appendix

| **Study variable** | **Definition** |
| --- | --- |
| Age at first sexual assault | Age at first known sexual aggression |
| Parents separated | Parents were separated at the time of the assessment |
| Out-of-home placement | Placement outside the family at the time of the assessment |
| Conduct disorder | Psychiatric diagnosis on file |
| ADHD | Psychiatric diagnosis on file |
| Low IQ | IQ > 55 and < 80, based on official data |
| Sexual violence | Participant was a victim of hand-on sexual assault |
| Physical violence | Participant was the victim of physical familial violence |
| Parental neglect | Participant was a victim of one of the following types of neglect: lack of supervision or of physical or sexual protection, permission for serious behavioural disturbance, medical neglect, failure to provide care, physical neglect |
| Isolation/social rejection | Participant is withdrawn or isolated from his peers |
| Deviant sexual fantasies | Participant admits to having had deviant sexual fantasies towards a victim or another person |
| Male victim | At least one male victim of sexual aggression on record in the participant’s file |
| Stranger victim | At least one documented case of a previously unknown victim of sexual aggression on file |
| Deviant sexual behaviours | Participant exhibited one of the following behaviours: fetishism, voyeurism, exhibitionism, compulsive masturbation, obscene phone calls, frottage, bestiality, sexual harassment |
| Criminal record | History of criminal record under the Loi sur les Jeunes Contrevenants (Young Offenders Act) |
| Delinquent peers | Participant associates with delinquent or dubious peers |
| Alcohol/drug consumption | Any self-reported consumption |
| Early-onset aggressivity | The participant exhibits a pattern of repeated verbal or physical violence, violence against objects, or animal cruelty before 12 years old. |
| Physical violence to peers | History of physical violence against peers |
